# LED-driven controlled deposition of Ni onto TiO_2_ for visible-light expanded conversion of carbon dioxide into C_1_–C_2_ alkanes†

**DOI:** 10.1039/d1na00021g

**Published:** 2021-04-20

**Authors:** Arturo Sanz-Marco, José L. Hueso, Víctor Sebastian, David Nielsen, Susanne Mossin, Juan P. Holgado, Carlos J. Bueno-Alejo, Francisco Balas, Jesus Santamaria

**Affiliations:** Department of Chemical and Environmental Engineering, University of Zaragoza c/Mariano Esquillor, s/n; Campus Rio Ebro, Edificio I+D Zaragoza 50018 Spain fbalas@unizar.es; Institute of Nanoscience and Materials of Aragon (INMA), University of Zaragoza, Consejo Superior de Investigaciones Científicas (CSIC) c/Mariano Esquillor, s/n 50018 Zaragoza Spain; Networking Research Center in Biomaterials, Bioengineering and Nanomedicine (CIBER-BBN) C/Monforte de Lemos, 3-5 28029 Madrid Spain; Centre for Catalysis and Sustainable Chemistry, Department of Chemistry, Technical University of Denmark Kemitorvet 207 2800 Kgs. Lyngby Denmark; Instituto de Ciencia de Materiales de Sevilla (ICMS, CSIC-University of Seville) Avda. Americo Vespucio, s/n Seville 41092 Spain

## Abstract

Photocatalytic gas-phase hydrogenation of CO_2_ into alkanes was achieved over TiO_2_-supported Ni nanoparticles under LED irradiation at 365 nm, 460 nm and white light. The photocatalysts were prepared using photo-assisted deposition of Ni salts under LED irradiation at 365 nm onto TiO_2_ P25 nanoparticles in methanol as a hole scavenger. This procedure yielded 2 nm Ni particles decorating the surface of TiO_2_ with a nickel mass content of about 2%. Before the photocatalytic runs, Ni/TiO_2_ was submitted to thermal reduction at 400 °C in a 10% H_2_ atmosphere which induced O-defective TiO_2−*x*_ substrates. The formation of oxygen vacancies, Ti^3+^ centers and metallic Ni sites upon photocatalytic CO_2_ hydrogenation was confirmed by *operando* EPR analysis. *In situ* XPS under reaction conditions suggested a strong metal–support interaction and the co-existence of zero and divalent Ni states. These photoactive species enhanced the photo-assisted reduction of CO_2_ below 300 °C to yield CO, CH_4_ and C_2_H_6_ as final products.

## Abstract 2

Photodeposited 5 nm Ni nanoparticles on TiO_2_ P25 catalysts promote the photocatalytic hydrogenation of CO_2_ under visible light to CH_4_ and C_2_H_6_.

## Graphical abstract

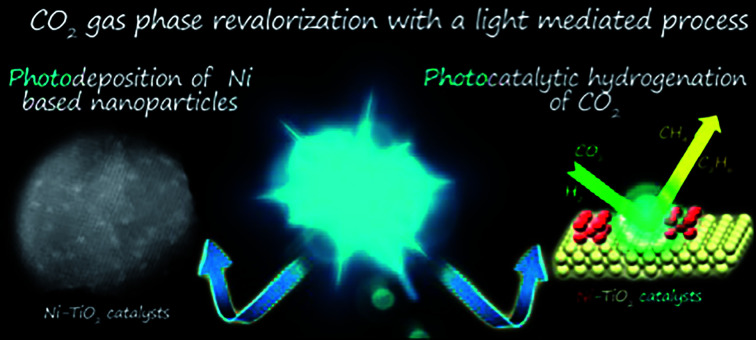

## Introduction

The overall depletion of fossil fuels and the overwhelming evidence of the impact of greenhouse gas emissions on the global environment are among the major challenges of the humankind today^1,2^ and demand a swift transition to renewable and sustainable processes. Among the strategies advocated to overcome these issues are gas-phase capture and storage, and revalorization by reduction to value-added products. In particular, the catalytic hydrogenation of CO_2_ for synthetic fuel production is an appealing mid-term solution to reduce the concentration of greenhouse gases, mitigate the dependence on fossil fuels and promote an overall neutral CO_2_ emission cycle.^3–7^

Photocatalysis involves a greener vision of chemical processes, including the smart and selective transformation of chemicals into fuels and/or chemical intermediates of interest.^8–10^ The use of photocatalysts leads to an effective depletion of organic pollutants, either in the form of wastewater or as organic volatile compounds.^11,12^ In fact, photocatalysis takes advantage of sunlight as an abundant and inexpensive energy source, providing a sustainable approach to solving the most pressing environmental issues. Alternatively, energy-efficient artificial light systems, such as light emitting diodes (hereafter LEDs) have revealed themselves as mighty allies for inducing photocatalytic processes with enhanced effectiveness (see for instance ref. 13). Current efforts are being devoted to developing novel photocatalysts that can exploit the full-solar spectrum.^14–18^

Most of the current photoactive materials are based on semiconducting oxides and heterojunctions,^19,20^ where light irradiation promotes the formation of an electron–hole pair, or an exciton (e^−^–h^+^), in the electronic band structure of the solid, followed by the redox reaction on the surface of the catalyst. Among them, titanium dioxide, TiO_2_, is the most studied photocatalytic semiconductor, due to its high photoactivity, low cost, natural abundance and non-toxicity.^21^ However, its wide band gap (*E*_g_ = 3.2 eV for the anatase phase) limits its photo-activity to the UV region.^22,23^ Some of the strategies to enhance the catalytic activity of TiO_2_ involve the addition of nanostructured co-catalysts for improving the charge separation and the catalytic kinetics^6,24^ or the controlled generation of structural defects to enhance the solar absorption and the gas species adsorption on the surface.^25^ Since the seminal work on TiO_2_ as a heterogeneous photocatalyst,^26^ there has been an outstanding development of semiconducting TiO_2_-based nanostructured materials, particularly for environmental remediation applications under both liquid^27,28^ and gas phase conditions.^29–31^

A wide variety of noble metal co-catalysts have been tested for the gas phase hydrogenation of CO_2_ into alkanes mediated by the Sabatier's reaction in the gas phase under diverse conditions.^32–35^ In addition, first-row elements have been deposited on the surface of nanosized TiO_2_ under analogous conditions to develop efficient photocatalysts for numerous environmental applications.^36^ Particularly, nickel on TiO_2_ exhibited a high activity for the methanation reaction with dependence on the morphology, crystalline state and metal–support interactions.^37^ The photo-assisted catalytic activity of Ni-containing TiO_2_ catalysts has been tested, which showed that the work function of Ni lies in the appropriate range (5.3 eV) to enhance the charge separation of the TiO_2_ electron bands.^38^ Furthermore, Meng *et al.* showed that even oxidized Ni species, such as Ni(OH)_2_, on TiO_2_ nanofibers were effective for the reduction of CO_2_ to methane and carbon monoxide.^39^ Similarly, Ni nanoclusters on reduced TiO_2_ supports have been reported to be effective for the photocatalytic conversion of CO_2_ to alcohols *via* an aldehyde mechanism.^40^ To deposit metal crystallites on TiO_2_ a variety of methods have been used, including hydrothermal, co-precipitation and thermal evaporation procedures, yielding metal nanoparticles with sizes down to 10 nm on the surface of TiO_2_.^41,42^

Recently, the photocatalytic nature of some semiconducting metal oxides has been leveraged to reduce metal ions to nanosized metal particles upon light irradiation.^43,44^ This so-called photodeposition procedure is a liquid-phase reaction that uses light in the UV range to induce an exciton pair in the surface of TiO_2_,^45^ which is first trapped by surface oxygen atoms. The photogenerated holes at the Ti–OH sites act as active species for the oxidation of solvent molecules, *e.g.* methanol, through hydrogen evolution, and the electrons cause the reduction of metal cations present in the liquid, which precipitate as metallic particles on the surface of TiO_2_.^46,47^ The hole scavenging process allows a longer life of the e^−^–h^+^ pair and therefore a more effective metal ion reduction and deposition is achieved. Usually, the photo-assisted deposition procedure leads to metal particles, with small sizes (*e.g.* 5 nm), on the semiconducting oxide.^42^

In the present work, Ni-based nanosized particles have been deposited on the surface of TiO_2_ nanoparticles by a photodeposition method in methanol as a hole scavenger. These materials have been successfully tested in the gas-phase photocatalytic hydrogenation of CO_2_ in a fixed-bed reactor under LED irradiation at different wavelengths, including the full visible spectrum. The generation of structural defects (*i.e.* oxygen vacancies and Ti^3+^ defective centers) within the TiO_2_ framework was investigated with *operando* EPR. *In situ* XPS studies under reaction conditions further elucidated the coexistence of metallic and divalent Ni domains partially buried or decorating the TiO_2_ support, thereby suggesting a strong metal–support interaction (SMSI). We found that the *ex situ* thermal pre-activation of the Ni–TiO_2_ strongly promoted the light-driven/light-assisted/LED-driven CO_2_ hydrogenation towards carbon monoxide, methane and ethane.

## Experimental section

### Synthesis of Ni–TiO_2_ by LED-assisted photodeposition

A photodeposition method was used to attach Ni nanoparticles on the TiO_2_ surface. Briefly, 25 mg of nanosized TiO_2_ (Evonik P25, Dusseldorf, Germany) and 55 mg of nickel chloride hydrate (NiCl_2_·6H_2_O 98%, Sigma-Aldrich) were added to 17 ml of DI water in a 20 ml Pyrex tube, which was then closed with a rubber septum. After 20 min of degasification under nitrogen gas flow, 1 ml of methanol (CH_3_OH, Sigma-Aldrich) was added to the mixture. All reagents and precursors were used without purification. The suspension was subsequently irradiated with UV light using two LED lamps (OSRAM LED Engin, Wilmington MA) at 365 nm for 270 s and under continuous magnetic stirring to induce the photocatalytic reduction of Ni salts. The so-obtained suspension was further centrifuged at 7500 rpm for 5 minutes, washed several times with a mixture of double-deionized water and ethanol, and dried at 100 °C for 10 h. The final solid samples were stored in the dark for further use and characterization. Finally, some of the samples were submitted to a gas-phase *ex situ* reduction procedure before characterization and photocatalytic activity tests. Typically, 150 mg of the catalyst were loaded into a ceramic crucible and placed in a tube furnace (Carbolite, Hope Valley, UK) under a 10 : 90 H_2_ : N_2_ atmosphere at 50 sccm and heated up to 400 °C at a 10 °C min^−1^ heating rate. The temperature was held for 2 h before cooling to room temperature under the same atmosphere. After thermal reduction, the TiO_2_-based nanoparticles developed a greyish color. These nanomaterials were prepared at the Synthesis of Nanoparticles Unit 9 of the ICTS “NANBIOSIS” at the Institute of Nanoscience of Aragon and the University of Zaragoza.

### Characterization

The crystalline structures of the TiO_2_ support and the photo-deposited Ni nanoparticles were identified by the X-ray diffraction (XRD) technique using a Bruker D8 Advance Diffractometer (Bruker Corporation, Billerica MA) equipped with a (002) Ge monochromator using the CuKα_1_ line at 1.5405 Å. The UV-vis spectra were recorded in a JASCO V-670 UV-vis/NIR spectrophotometer (JASCO, Tokyo, Japan) using the solid-state diffuse reflectance technique in a 60 mm UV-vis/NIR integrating sphere from 200 nm to 900 nm, with a scanning step of 10 nm s^−1^. Temperature-programmed reduction (TPR) tests were performed in a Quantachrome ChemBET Pulsar TPR/TPD analyzer (Quantachrome Instruments, Boynton Beach, FL) equipped with a Thermal Conductivity Detector (TCD). Usually, 50 mg powdered samples were loaded in one of the branches of a U-tube quartz reactor suspended between quartz wool pieces. Before every TPR test, samples were heated up to 100 °C under Ar gas flow to remove adsorbed species on the material surface. After cooling to room temperature, samples were heated from room temperature to 900 °C at 10 °C min^−1^ heating rate under 10 : 90 H_2_/Ar gas flow at 20 sccm. The BET surface area and porosity of catalysts were determined by means of N_2_ adsorption at 77 K using a Micromeritics TriStar 3000 analyzer (Micromeritics, Norcross, GA).

The TiO_2_ P25 and Ni/TiO_2_ were investigated by *in situ* and *operando* Electron Paramagnetic Resonance spectroscopy (EPR) on a Bruker EMX EPR instrument fitted with a ST4102 cavity and a Bruker variable temperature unit and quartz insert. The materials were pressed into pellets, then crushed and fractioned (150–300 μm). ∼25 mg of sample were immobilized with quartz wool in a 4 mm inner diameter quartz tube and placed in the cavity. The EPR spectra (9.45 GHz, 20 mW, 5 Gauss modulation amplitude at 100 kHz) were continuously obtained while introducing a total gas flow of 50 Nml min^−1^ during the sample analysis. To study the influence of light irradiation, UV light pulses in 100 s intervals (Dymax Blue Wave 75) were directed *via* a glass fiber to shine on the sample tube through a grid on the side of the EPR cavity. The same experimental protocol (see the ESI†) was performed on P25 and on Ni/TiO_2_ while monitoring with EPR to compare the responses of the photocatalysts under UV light exposure and different reaction atmospheres (see Table S1 in the ESI†). Samples were first activated in 30% H_2_ at 250 °C. Then the influence of UV light and of the H_2_/CO_2_ gas (separately and together) was investigated at room temperature. Then the sample was reactivated and the procedure was repeated at 250 °C. In general, the spectra exhibited lower resolution at 250 °C, as the noise level was higher at these temperatures and the changes were more difficult to discern. Overall, the same evolution was observed, but it was less clear and thus only room temperature spectra are shown in the text. The spectrum assigned to Ni species (type 1, see the main text) changed with temperature.

The Ni metal loading was evaluated using a microwave plasma-atomic emission spectrometer (4100 MP-AES, Agilent) and X-ray photoelectron spectroscopy (XPS) spectra were obtained before and after activation using a Kratos AXIS Ultra DLD surface analysis spectrometer (Kratos Analytical Ltd., Durham, UK). *In situ* XPS studies were performed using a customized system incorporating a hemispherical analyzer (SPECS Phoibos 100), a non-monochromatized X-ray source (Al Kα; 1486.6 eV, Mg Kα, 1253.6 eV) and a high temperature–high pressure cell (SPECS-HPC-20) that allows sample heating and gas flow. The analyzer was operated at a fixed transmission and 50 eV pass energy with an energy step of 0.1 eV. Binding energies were calibrated using C 1s or Ti 2p (284.6 eV or 458.8 eV) as an internal reference. The high temperature–high pressure cell design allowed sample heating up to 800 °C, under flow or static conditions, at pressures up to 20 bar or dynamic flows in the range 20–500 Nml min^−1^. This arrangement enabled a transfer of post-reaction samples from the reaction chamber to the spectrometer under UHV conditions, avoiding exposure to the laboratory atmosphere. Prior to each analysis, the samples were evacuated to 10^−9^ mbar at room temperature. In a typical experiment, the sample was initially placed in the sample holder (in the form of a pelletized disc) and transferred to the spectrometer chamber where XPS spectra were acquired. The sample was then transferred under vacuum to the high-pressure cell where it was exposed to the reactive gases and heated to the appropriate temperature. The flow and concentration of gases were similar to those used for catalytic testing (*vide infra*). After the treatment the sample was cooled to room temperature under the reaction atmosphere, evacuated down to 10^−7^ mbar in less than two minutes and then transferred back to the spectrometer chamber for analysis, avoiding ambient exposure.

Aberration corrected scanning transmission electron microscopy (Cs-corrected STEM) images were acquired using a high angle annular dark field detector in an FEI XFEG TITAN electron microscope operated at 300 kV equipped with a CETCOR Cs-probe corrector from CEOS Company allowing the formation of an electron probe of 0.08 nm. The geometric aberrations of the probe-forming system were controlled to allow a beam convergence of 24.7 mrad half-angle to be selected. Elemental analysis was carried out with an EDX detector for EDS experiments in scanning mode. EDX mapping was performed with an Oxford Instruments Detector and analysed with AZtec software provided by the detector manufacturer.

### Photo-assisted carbon dioxide hydrogenation tests

**Scheme 1:**
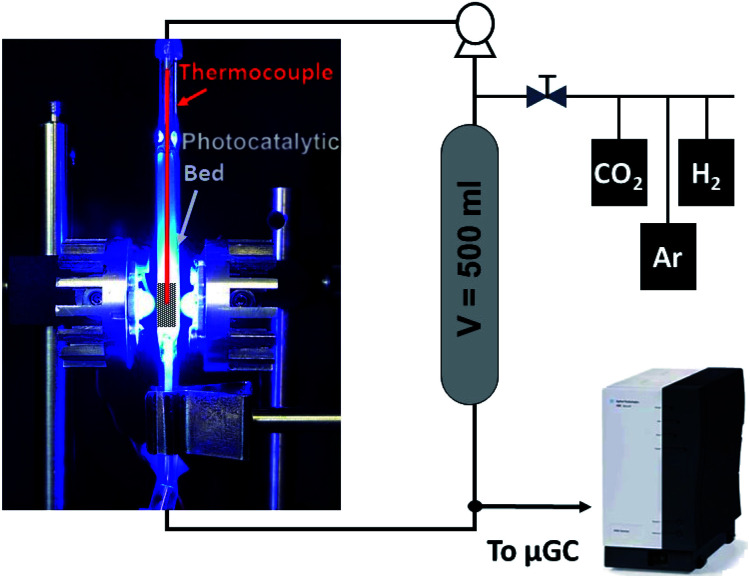
Simplified flowchart of the batch reactor system used for the photocatalytic tests. Typically, the batch reactor was filled with a 25 : 75 CO_2_/H_2_ mixture until a *P* = 1.7 bar was obtained. A 15 Nml min^−1^ gas flow was recirculated through the reactor. The fixed bed was operated under 9 W LED light (4.5 W per LED). A sample of the gas mixture was analyzed by gas chromatography hourly.

The CO_2_ hydrogenation was conducted in a fixed-bed reactor as shown in Scheme 1. Typically, 120 mg of powdered catalyst was packed in a 30 × 10 × 2 mm prismatic quartz reactor that was positioned between two LED sources of different wavelengths, namely 365 nm, 460 nm and white light. Before irradiation, a gas mixture of CO_2_ and H_2_ with a 1 : 4 molar ratio was fed into the reactor for 30 min to ensure air removal from inside the reactor. After that, the reactor was closed and filled with the gas mixture until an absolute pressure of 1.7 bar was reached. A recirculating OEM pump (Verderflex M1500, Groeningen, NL) caused a 7.5 ml min^−1^ gas flow through the reactor circuit that included a 500 ml chamber to buffer pressure oscillations and provide enough total volume for gas sampling in prolonged experiments. The reaction was started by turning on the LEDs, which also caused a sharp increase in the solid temperature to reach a stable value around 250 °C. A sample of the gases was periodically analyzed using an on-line 490 micro-GC analyzer (Agilent Technologies, Santa Clara, CA, equipped with three columns: a 10 m molecular sieve column with a 5 m PPQ precolumn, a 10 m PBQ column and a 10 m CP-wax). DC current was set at 0.9 A and 12 V during LED irradiation for all tested materials.

## Results and discussion

### Photodeposition of Ni nanoparticles on TiO_2_ P25

The photo-assisted deposition method involves the photonic excitation of a semiconductor immersed in a solution that contains the ionic precursor of the material to be deposited and at least one liquid component that can act as a hole scavenger. When the incident photon energy is larger than the *E*_g_, the valence band electrons can be excited to the conduction band.

**Fig. 1:**
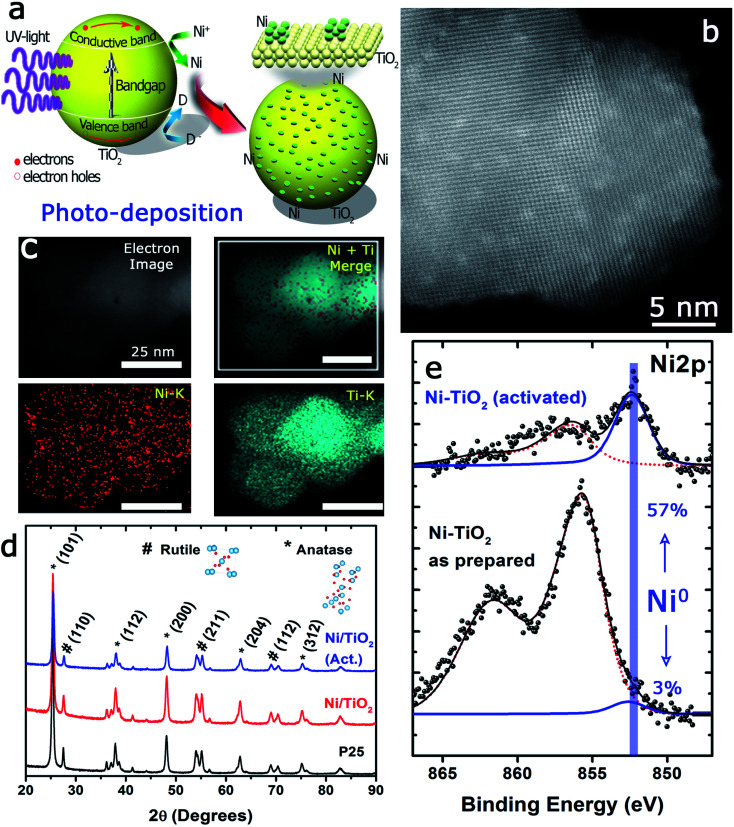
Characterization of the Ni–TiO_2_ photocatalyst: (a) scheme of the photo-assisted deposition process; (b) HAADF-STEM representative image of the Ni–TiO_2_ photocatalyst. Dots displaying lighter contrast correspond to the small Ni domains homogeneously dispersed on the TiO_2_ support; (c) EDX images corresponding to the selected areas shown in the inset STEM image accounting for the presence of Ti and Ni domains altogether in the catalyst nanoparticles; (d) XRD patterns of TiO_2_ nanoparticles after photodeposition of Ni from NiCl_2_ as the precursor and CH_3_OH as the hole-scavenging solvent. Crystalline Ni species remained undetected and only the reflections of anatase and rutile could be found. The activation procedure at 400 °C in a 10% H_2_ atmosphere reduced the relative height of the (100) reflection of anatase together with a slight increase in the peak width; (e) XPS spectra corresponding to the Ni 2p_3/2_ region before and after activation in a reducing environment. A strong increase of reduced Ni species is observed after activation.

The pumped electrons are therefore used for the reduction of the metallic cations in the electrolyte, Ni^2+^ in this case, to deposit nanosized metallic particles on the surface of the semiconducting oxide. To avoid fast electron–hole pair recombination or the oxidation of the photodeposited metallic particles, methanol, an easy to oxidize substrate, is used, which acts as a sacrificial agent and hole scavenger when added to the electrolyte (Fig. 1a). The photo-deposition process provided white solids with a Ni mass concentration of 1.10 ± 0.11% calculated by MP-AES spectrometry (see the Experimental section). The subsequent thermal activation at 400 °C in a 10%-H_2_ atmosphere produced light grey-colored solids with a Ni mass concentration of 1.18 ± 0.05%, *i.e.*, thermal activation in the hydrogen atmosphere did not alter the Ni loading of the prepared catalysts. The Ni/TiO_2_ catalyst particles exhibited similar size before and after thermal activation, between 20 and 25 nm. High-resolution HAADF-STEM images of individual Ni/TiO_2_ nanostructures showed 2 nm Ni/NiO crystallites homogeneously dispersed on the TiO_2_ nanoparticles (Fig. 1b). EDX analysis of individual nanoparticles confirmed the presence of Ni domains (see Fig. 1c). XRD analysis (Fig. 1d) showed the blend of anatase and rutile diffraction peaks expected for TiO_2_ P25 nanoparticles, without any diffraction pattern attributable to Ni or nickel oxides. This might be attributed to the concentration of deposited Ni (<2 wt%), particularly to its extraordinary dispersion and small particle size. These 2 nm sized Ni particles could be assigned to either Ni or NiO nanoparticles. Two different chemical processes could explain the presence of NiO after photodeposition: oxidation of the deposited Ni^0^ nanoparticles can take place upon environmental exposure, or the direct photodeposition of NiO particles may take place in a methanol-deficient medium.^48,49^

**Fig. 2:**
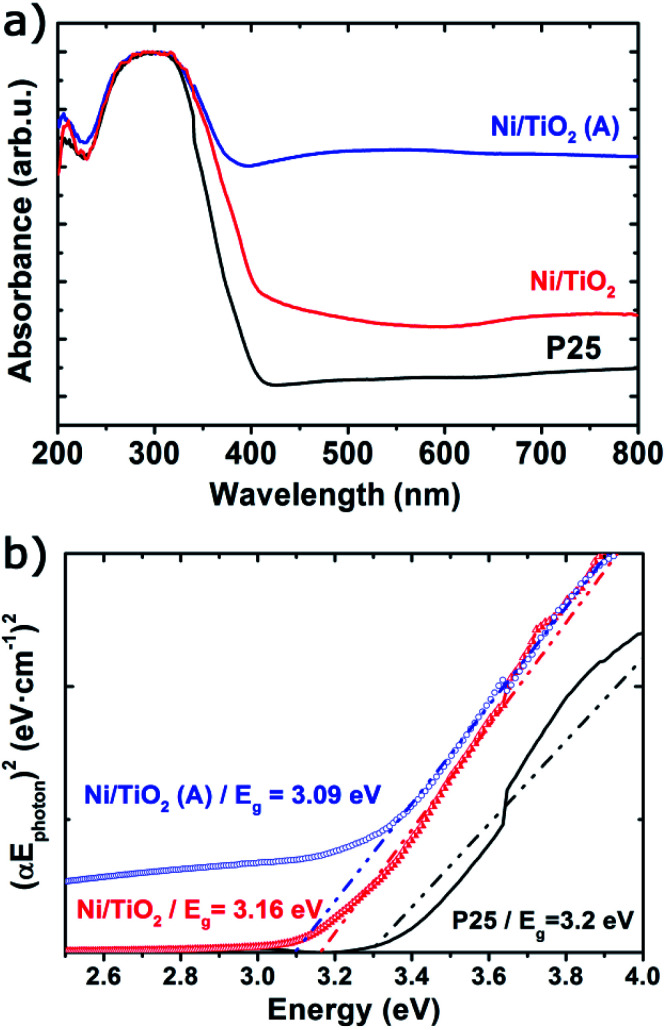
UV-vis absorbance spectra (a) and Tauc plots (b) of TiO_2_ before and after Ni photodeposition in MeOH/H_2_O (Ni/TiO_2_) and thermal activation at 400 °C in a 5%-H_2_ atmosphere (Ni/TiO_2_-A).

The UV-vis spectra showed very significant changes with respect to the absorption bands of TiO_2_ P25 (Fig. 2a), which displayed a strong absorption below 400 nm corresponding to the ground excitonic state of TiO_2_. The absorption bands of Ni/TiO_2_ were expanded towards the visible–near infrared region resulting from the formation of either metallic Ni or NiO nanoparticles.^42,50,51^ After thermal activation, the absorption in the visible region is greatly enhanced, which is due to the reducing conditions that led to the formation of oxygen vacancies and the incorporation of H-containing species or even Ti^3+^ cations on the surface of TiO_2_.^52,53^ Such structural modifications induced the formation of intermediate energy levels in the band gap, leading to broader light absorption.^25^ Furthermore, the optical band gap estimated using the Tauc plots (Fig. 2b) returned a value of *E*_g_ = 3.2 eV for TiO_2_, in good agreement with values reported elsewhere.^23,25^ For Ni/TiO_2_, a red-shift in the optical band gap could be observed which can be ascribed to an interfacial charge transfer process, consisting in the migration of electrons from the semiconducting substrate to the metallic nanoparticles directly from the valence band after excitation.^54,55^ This feature was slightly more noticeable after the thermal activation in a reducing atmosphere, which could be due to the formation of O^2−^ vacancies or Ti^3+^ species on the TiO_2_ surface under those conditions.^56^

**Fig. 3:**
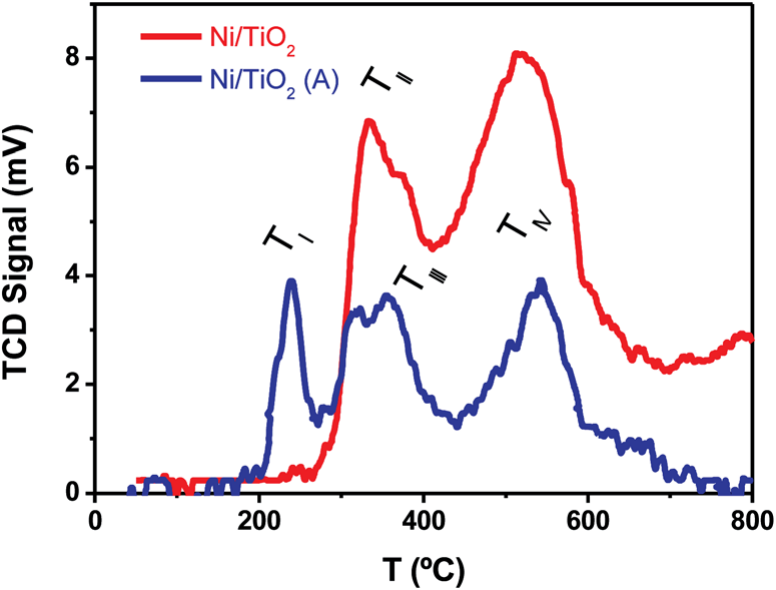
TPR plots of TiO_2_ before and after Ni photodeposition in MeOH/H_2_O, as prepared (Ni/TiO_2_), and after thermal activation at 400 °C in a 10%-H_2_ atmosphere (Ni/TiO_2_-A).

The TPR tests (Fig. 3) showed that Ni^2+^, presumably as NiO, and Ni^0^ were both likely present on Ni/TiO_2_ and Ni/TiO_2_-A. This was concluded after the detection of reduction peaks at *T*_II_ ∼ 330 °C and *T*_III_ ∼ 370 °C which were attributed to the reduction of NiO species with low and high interaction with the support, respectively.^57^ The signal centered at *T*_IV_ ∼ 550 °C was assigned to the reduction of smaller NiO nanoparticles dispersed in the TiO_2_ substrate with a very strong interaction.^50,58^ A noticeable decrease in the signal intensities was observed for the Ni/TiO_2_-A, which was attributed to the partial reduction of the Ni-based particles and TiO_2_ substrate after the thermal activation in a 10%-H_2_ atmosphere. However, the appearance of a signal at *T*_I_ ∼ 230 °C after the activation suggested the presence of NiO particles with a weak or null interaction with the support,^58^ which is in agreement with the decrease in Ni loading observed by XPS (Table 1 and Fig. S1†). This decrease in Ni loading is also observed after a photocatalytic evaluation, due to the stronger reduction atmosphere of photocatalytic tests (80%-H_2_), which could lead to a better integration of Ni clusters within the TiO_2_ surface.

**Table 1:** Binding energies (BE, eV) and surface concentration (at%) obtained by XPS analysis for the as-prepared and activated Ni/TiO_2_ catalysts before and after LED-assisted gas-phase photocatalytic CO_2_ hydrogenation

BE (eV), surface concentration (at%)				
	O 1s	Ti 2p	Ni 2p	C 1s
Ni/TiO_2_	530.0	458.8	856.0	285.0
	48.99%	21.69%	3.06%	26.26%
Ni/TiO_2_ (post-reaction)	530.3	458.3	856.3	285.3
	51.69%	20.33%	1.03%	26.94%
Ni/TiO_2_-A	529.8	458.6	855.6	285.0
	48.50%	23.92%	0.66%	26.92%
Ni/TiO_2_-A (post-reaction)	529.4	458.4	855.4	284.4
	51.45%	21.63%	0.14%	26.78%

### *In situ* and *operando* spectroscopic studies under CO_2_ hydrogenation conditions

With the aim of discerning the precise chemical state of the Ni and Ti on the surface of the catalyst, *operando* EPR spectroscopy has been used. Since both Ni and Ti paramagnetic centers, as well as potential ion defects, are present on the surface of the catalysts, EPR spectroscopy is undoubtedly a technique of choice.^59^ For instance, Morra *et al.* have used EPR to discern the active catalytic sites in Ti-containing materials, where titanium ions could be formed in low oxidation states under diverse reaction conditions.^60^

**Fig. 4:**
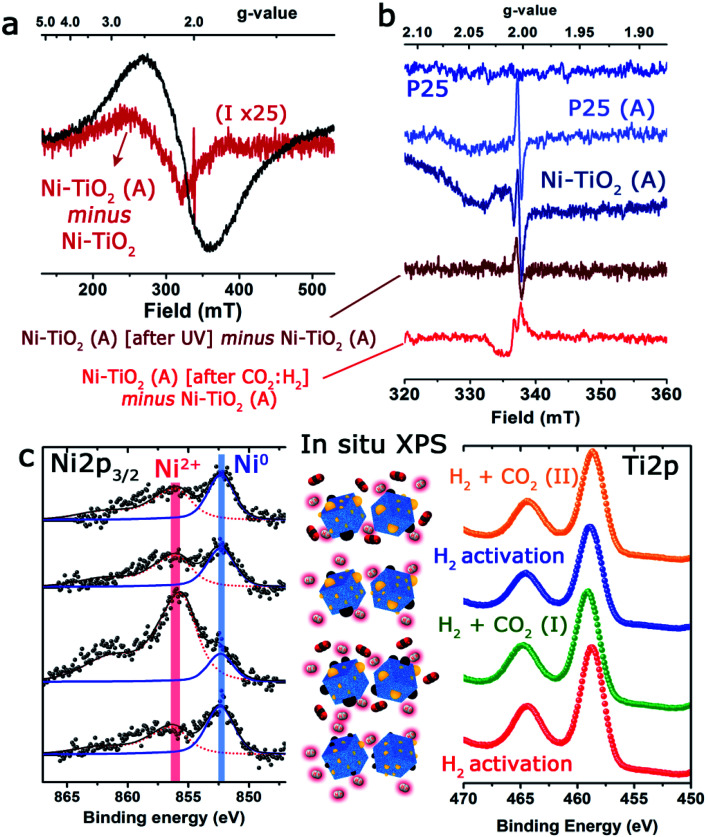
*In situ* and *operando* spectroscopic studies under a CO_2_ hydrogenation reaction environment: (a) selected X-band EPR spectra collected at room temperature of TiO_2_ and Ni/TiO_2_ before and after thermal activation at 250 °C in 30% H_2_ and of Ni/TiO_2_-A before and after exposure to UV light and H_2_/CO_2_ at room temperature; full spectrum of Ni/TiO_2_ (black) and change in the EPR spectrum (×25) upon activation (red). (b) Zoom-in of the EPR spectra of TiO_2_ before (blue) and after activation (light blue), Ni/TiO_2_ after activation (dark blue) and the change after exposure to UV-light (brown) and after exposure to H_2_ + CO_2_ (light red); (c) XPS spectra corresponding to the Ni 2p_3/2_ and the Ti 2p regions after submitting the catalyst sample to successive thermal treatments for either the reduction/activation (10 vol% H_2_–Ar; flow rate: 20 Nml min^−1^; 400–450 °C) or carbon dioxide hydrogenation reaction (CO_2_ : H_2_ – 1 : 4; flow rate: 5 Nml min^−1^; 60 mg of pelletized 1.2 wt% Ni/P25 photocatalyst; 225 °C).

In the present study, three different types of EPR signals were observed when performing the *in situ* and *operando* protocol. Fig. 4 shows a selection of EPR spectra and the most relevant changes observed under reaction conditions. Type 1 is observed for Ni/TiO_2_, but not for P25 TiO_2_. It is a huge broad peak centered at *g* ∼ 2.1 (Fig. 4a), constantly present for Ni/TiO_2_ but changes during activation, and is unaltered upon exposure to UV light. In order to follow the subtle change during activation, the spectra of the fresh sample were subtracted from the spectrum of the activated sample (Fig. 4a).

The spectral change corresponded to a broad EPR spectrum with a *g*-value of ∼2.34 and a tiny sharp peak at 2.0035. The *g*-value of the broad signal in the difference spectrum was significantly shifted compared to the *g*-value of the overall spectrum (∼2.1). Type 1 was assigned to paramagnetic Ni centers or defects in the Ni nanoparticles, which suggests the contribution from several types of Ni species, which could be either Ni^2+^ or Ni^+^. The EPR spectrum is largely featureless for a precise assignment, but it is noted that the *g*-value of 2.34 for the evolved species is similar to the value observed for defect sites in pure NiO nanoparticles.^61^ The type 2 signal was a small sharp isotropic signal centered at 2.0035, which was absent for the fresh materials but showed up after a few minutes and in comparable intensities for both TiO_2_ and Ni/TiO_2_ after activation (heating to 250 °C in 30% H_2_). The signal of Ni/TiO_2_-A increased during exposure to UV light (still under H_2_), but returned back to the initial state after the light was turned off (Fig. 4b). No clear development in the signal was observed for TiO_2_ during interaction with UV light (data not shown). The signal is not influenced by either of the materials being exposed to the H_2_/CO_2_ gas mixture. The type 2 signal was assigned to sites in the bulk of the nanoparticles: either an F-center, an electron caught in an oxygen vacancy, or a V-center, an oxygen ion caught at a cation vacancy.^62,63^

Finally, type 3 was a small anisotropic signal centered at 2.011 which is visible only for Ni/TiO_2_-A. It overlapped with the type 1 signal and was barely influenced by exposure to UV light; however, it disappeared completely after exposure to the H_2_/CO_2_ mixture (notice the negative change in the spectrum in Fig. 4b). It was tentatively assigned to oxygen-centered surface hole trapping sites in the anatase polymorph of TiO_2_ P25.^64^ The anisotropy of the signal is due to the direction dependence of surface sites. This type of site is observed in the literature on pure TiO_2_ at low temperatures (<100 K) where the signal to noise ratio of EPR is much better. In our catalyst such evidence suggests that the introduction of very small Ni particles by photodeposition has promoted the number of these sites to the extent that they are now observable by EPR at room temperature and above. The fact that they are sensitive to the H_2_/CO_2_ mixture confirms the assignment as surface sites and is an indication that they are involved directly in the reaction of CO_2_ on the surface of the material.

The state of Ni species on the TiO_2_ substrates was further explored using XPS analysis (Fig. 4c and Table 1). It is interesting to note that the XPS signals corresponding to the Ni 2p levels were rather complex, being deconvoluted up to three Gaussian/Lorentzian peaks (see the ESI, Table S2 and Fig. S1†). This situation is typically found in the presence of multiplet contributions and satellite structures, whose formation could be found in recent papers.^50,65^ For Ni^2+^ the satellite at about +6.0 eV might be assigned to a final state effect associated with a (core) 3d^8^L configuration (where L stands for ligands). However, the intensity and position of this satellite depended on many factors such as bonding with ligands, symmetry, particle size and crystallinity.^66,67^

Furthermore, a noticeable decrease of the atomic concentration of Ni was observed after thermal activation under a H_2_ atmosphere at 400 °C (Table 1). This might be attributed to the process of Ni nanoparticle growth by sintering or, alternatively, to an incomplete immersion of the Ni particles into the support due to the “decoration” effect of the TiO_2_ over the Ni particles. This has been previously reported for a variety of metal catalysts on different supports,^68^ within the context of the strong metal–support interaction (SMSI) effect.^69^ Both events would be enhanced in the case of nanosized particles^70,71^ and under treatment with hydrogen. In addition, the absence of complete reduction of Ni particles (only 57% of Ni^0^ after the reduction treatment) would also support this hypothesis.

*In situ* XPS observations were carried out to evaluate the evolution of the oxidation states of Ni and Ti under activation or CO_2_ hydrogenation conditions at 225 °C giving interesting and complementary insights into the *operando* EPR results. Although the change of the oxidation state of Ti was unclear (Fig. 4c), after the initial CO_2_/H_2_ treatment, an incomplete recovery of Ni signal intensity and a notable oxidation of the particles were noticed (Ni^2+^ concentration about 84%). These facts would be in agreement with the expected behavior in a SMSI scenario, with the CO_2_ serving as a mild re-oxidation agent. After the second reduction treatment at 450 °C, the situation was similar to that described before, and a decrease in intensity and a partial reduction of the Ni particles were noticed (Fig. 4c and Table S3†).

Nevertheless, after the second treatment with the CO_2_/H_2_ mixture, neither the Ni signal intensity nor the Ni^2+^/Ni^0^ ratios were remarkably altered. This could reflect a more permanent coverage of the Ni particles caused by the reduction at a higher temperature under hydrogen and would reinforce the close interaction between Ni and TiO_2_ as suggested by EPR. Actually, the HAADF-STEM images of the catalysts after reaction revealed that while a fraction of Ni domains have coarsened, the majority of the catalyst maintained similar particle size distributions (see Fig. S2†).

### Light-assisted gas-phase photocatalytic CO_2_ hydrogenation

**Fig. 5:**
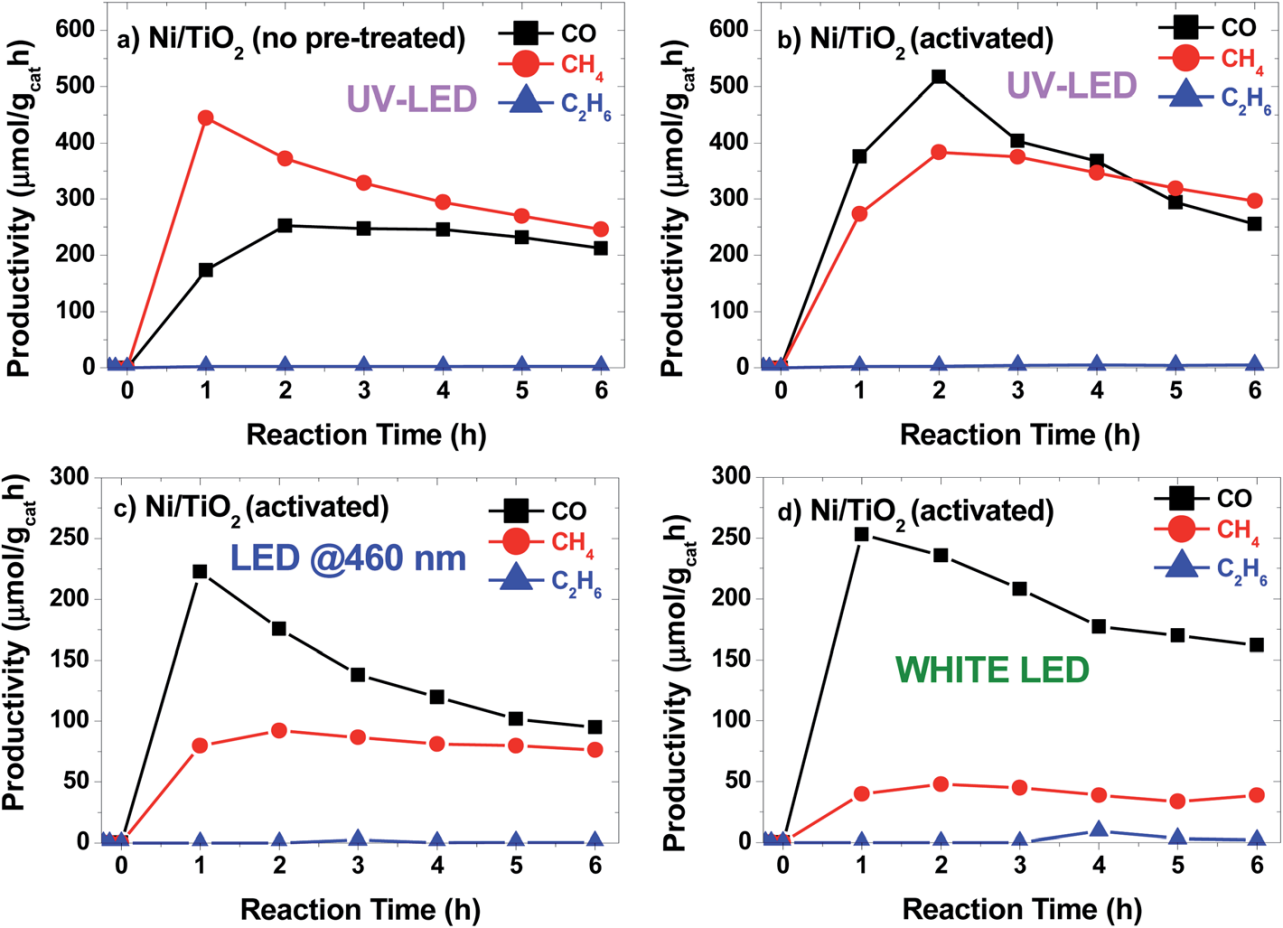
Productivity *vs.* time plots for Ni/TiO_2_ catalysts (a) under LED light irradiation at 365 nm and for Ni/TiO_2_-A catalysts under LED irradiation at 365 nm (b), 460 nm (c) and white light (d).

The photocatalytic conversion of CO_2_ into CO, CH_4_ and C_2_H_6_ alkanes was observed under LED irradiation at different wavelengths (Fig. 5). The photocatalytic performance of TiO_2_ P25 under UV light was evaluated before Ni photodeposition (see Fig. S4a†). Also, a control experiment was carried using Ni/TiO_2_ under 365 nm LED irradiation in 4/1 Ar/H_2_ stream. No CO, CH_4_ or C_2_H_6_ signals were detected after reaction for 6 h (Fig. S4b†). Results showed CO productivity around 100 μmol g_cat_^−1^ h^−1^ during the 6 h of reaction time. The signals attributed to both CH_4_ and C_2_H_6_ were also detected, at concentrations below the quantification limit. In contrast, when Ni/TiO_2_ catalysts were illuminated at 365 nm (Fig. 5a), the main product obtained was CH_4_ with a maximum initial productivity of 450 μmol g_cat_^−1^ h^−1^ and an increased CO productivity of 250 μmol g_cat_^−1^ h^−1^. Also, the production of C_2_H_6_ remained almost constant during the reaction, reaching about 2 μmol g_cat_^−1^ h^−1^. Catalyst activation by the high temperature hydrogen treatment produced two main effects: first, the photocatalytic activity increased significantly under UV light (Fig. 5b), and second, the catalytic response is expanded to the visible region of the solar spectrum (Fig. 5c and d). This increase in the CO and C_2_H_6_ (∼4 μmol g_cat_^−1^ h^−1^) productivity under UV light for the Ni/TiO_2_-A could be attributed to the formation of both bulk and surface oxygen vacancies (V_O_) on the TiO_2_ phase upon hydrogenation and the enhancement of the SMSI effect as suggested by the *in situ* XPS data. These V_O_ induced donor intermediate energy levels in the bandgap, which might act as electron traps, facilitating charge-carrier separation and charge transfer to the adsorbed species. The irradiation under UV LEDs was more effective for the generation of CO. A similar trend with lower productivity was also found for the Ni/TiO_2_-A under LED illumination at blue light (460 nm, Fig. 5c) and white light (Fig. 5d). Interestingly, similar catalytic activity tests under blue or white LED irradiation using the as-synthesized Ni/TiO_2_ catalysts before thermal activation showed CO productivity lower than 25 μmol g_cat_^−1^ h^−1^ up to 4 h of reaction (data not shown).

The temperature at the center of the fixed bed during the reaction was about 250 °C when 460 nm and white LED units were used, but increased to 270 °C and 300 °C for the Ni–TiO_2_ and Ni/TiO_2_-A under UV illumination, respectively, which could simply be a result of a higher absorbance of the catalyst at 365 nm, but may also reflect the contribution of plasmonic effects in the Ni/NiO nanoparticles located at the surface of the TiO_2_,^72,73^ as recent studies have shown that plasmonic nanoparticles deposited on TiO_2_ could act as nanoheaters on the surface of the semiconductor.^74^

**Fig. 6:**
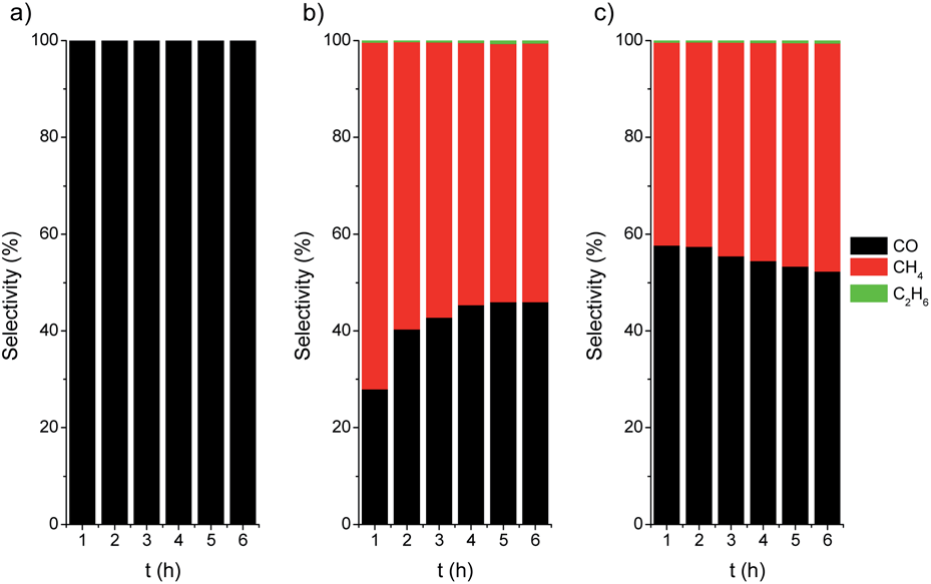
Selectivity *vs.* time plots for TiO_2_ P25 (a), Ni/TiO_2_ (b) and Ni/TiO_2_-A (c) catalysts under UV LED irradiation (365 nm).

The photothermal CO_2_ hydrogenation is expected to proceed through the exothermic Sabatier reaction (CO_2_ + 4H_2_ → CH_4_ + 2H_2_O, Δ*H*^0^ = −165 kJ mol^−1^).^75^ Alternatively, for TiO_2_-based materials the reverse water gas shift (RWGS) reaction has also been proposed, yielding CO and water vapor (CO_2_ + H_2_ → CO + H_2_O, Δ*H*^0^ = 41 kJ mol^−1^).^74^ It has been suggested that these reactions could occur through the dissociation of physisorbed CO_2_ into CO intermediates that either evolve to formate^76^ or to carbide to finally form CH_4_ molecules upon further hydrogenation.^77^ In our case the CO_2_ hydrogenation progressed to the formation of CH_4_ on active centers, namely Ni/NiO nanoclusters, followed to a certain extent by a C–C coupling up to the formation of C_2_H_6_. At this point, it should be noted that the direct formation of ethane from CO_2_ over Ni was reported to be very minimal.^78^ On the other hand, the C–C coupling in the gas phase to generate higher hydrocarbons, such as ethane, over Ni-based catalysts was recently reported to be independent of the structure and only controlled by the hydrogenation rate.^79^ The photo-assisted deposition of Ni was critical for the CO hydrogenation to alkanes (Fig. 6). For Ni-containing catalysts, the initial selectivity to CH_4_ is close to 70%, decreasing down to 60% after 6 h of catalytic test. After activation (Ni/TiO_2_-A), the selectivity slightly evolved towards CH_4_, which can be attributed to an increase in the SMSI after the reduction treatment.

Finally, the thermally activated catalysts (Ni/TiO_2_-A) also exhibited a higher production of both C_2_ and C_1_ alkanes. In this case, the formed V_O_ in the TiO_2_ could act as centers for the enhanced adsorption of CO_2_, thus improving the hydrogenation rate. The oxygen-defective TiO_2_ also induced the formation of Ti^3+^ species (see TPR data in Fig. 3), which have been reported to have a photonic response upon illumination with UV photons.^25,80,81^ These combined effects resulted in enhanced conversion of CO_2_ even under illumination in the visible range.

## Conclusions

Ni/NiO nanosized particles were effectively deposited on the surface of TiO_2_ nanoparticles by a photodeposition procedure that used Ni^2+^ salts as precursors and CH_3_OH as a hole scavenger. The synthesis of very small Ni crystallites on TiO_2_ was achieved under the irradiation of LEDs at 365 nm. A thermal activation under a reducing atmosphere allowed the stabilization of Ni^0^ on the catalyst surface, as well as the formation of oxygen vacancies that could enhance the CO_2_ adsorption.

Under LED illumination at different wavelengths, the Ni/TiO_2_ catalysts induced the photocatalytic hydrogenation of CO_2_ at 300 °C to produce CO, CH_4_ and C_2_H_6_ with a productivity of about 450 μmol g_cat_^−1^ h. The thermally reduced catalysts (Ni/TiO_2_-A) showed enhanced photocatalytic response in the UV region and higher CO productivities in all the studied wavelengths. As the thermal reduction induces the formation of active sites in the TiO_2_ support, the RWGS reaction is favored, increasing the CO selectivity. Further EPR and XPS analyses suggested that the close interaction of the metal and support plays an important role in the enhancement of photoactivity.

## Conflicts of interest

There are no conflicts to declare.

## Supplementary Material

NA-003-D1NA00021G-s001

## References

[cit1] IEA , World Energy Outlook 2018, IEA, Paris, 2018, https://doi.org/10.1787/weo-2018-en

[cit2] BP p.l.c. , BP Energy Outlook, 2019

[cit3] Li K., An X., Park K. H., Khraisheh M., Tang J. (2014). Catal. Today.

[cit4] Yaashikaa P. R., Senthil Kumar P., Varjani S. J., Saravanan A. (2019). J. CO_2_ Util..

[cit5] Wang L., Ghoussoub M., Wang H., Shao Y., Sun W., Tountas A. A., Wood T. E., Li H., Loh J. Y. Y., Dong Y., Xia M., Li Y., Wang S., Jia J., Qiu C., Qian C., Kherani N. P., He L., Zhang X., Ozin G. A. (2018). Joule.

[cit6] Wang Y., Zhao J., Li Y., Wang C. (2018). Appl. Catal., B.

[cit7] Zhang F., Li Y. H., Qi M. Y., Tang Z. R., Xu Y. J. (2020). Appl. Catal., B.

[cit8] Liu X., Duan X., Wei W., Wang S., Ni B. J. (2019). Green Chem..

[cit9] Mu Q., Zhu W., Li X., Zhang C., Su Y., Lian Y., Qi P., Deng Z., Zhang D., Wang S., Zhu X., Peng Y. (2020). Appl. Catal., B.

[cit10] Xu F., Zhang J., Zhu B., Yu J., Xu J. (2018). Appl. Catal., B.

[cit11] Tursunov O., Kustov L., Kustov A. (2017). Oil Gas Sci. Technol..

[cit12] Li X., Yu J., Jaroniec M., Chen X. (2019). Chem. Rev..

[cit13] Bueno-Alejo C. J., Hueso J. L., Mallada R., Julian I., Santamaria J. (2018). Chem. Eng. J..

[cit14] Zhang C., Liang H. Q., Xu Z. K., Wang Z. (2019). Adv. Sci..

[cit15] Ye R. P., Ding J., Gong W., Argyle M. D., Zhong Q., Wang Y., Russell C. K., Xu Z., Russell A. G., Li Q., Fan M., Yao Y. G. (2019). Nat. Commun..

[cit16] Liu Y., Miao C., Yang P., He Y., Feng J., Li D. (2019). Appl. Catal., B.

[cit17] Zhu S., Chen X., Li Z., Ye X., Liu Y., Chen Y., Yang L., Chen M., Zhang D., Li G., Li H. (2020). Appl. Catal., B.

[cit18] Feng X., Pan F., Zhao H., Deng W., Zhang P., Zhou H. C., Li Y. (2018). Appl. Catal., B.

[cit19] Yu X., Marks T. J., Facchetti A. (2016). Nat. Mater..

[cit20] Liras M., Barawi M., De La Peña O'Shea V. A. (2019). Chem. Soc. Rev..

[cit21] Dhakshinamoorthy A., Navalon S., Corma A., Garcia H. (2012). Energy Environ. Sci..

[cit22] Wang B., Shen S., Mao S. S. (2017). J. Mater..

[cit23] Liu Y., Tian L., Tan X., Li X., Chen X. (2017). Sci. Bull..

[cit24] Ortega-Liebana M. C., Hueso J. L., Ferdousi S., Arenal R., Irusta S., Yeung K. L., Santamaria J. (2017). Appl. Catal., B.

[cit25] Jia J., Qian C., Dong Y., Li Y. F., Wang H., Ghoussoub M., Butler K. T., Walsh A., Ozin G. A. (2017). Chem. Soc. Rev..

[cit26] Nakata K., Fujishima A. (2012). J. Photochem. Photobiol. C Photochem. Rev..

[cit27] Gaya U. I., Abdullah A. H. (2008). J. Photochem. Photobiol. C Photochem. Rev..

[cit28] Colmenares J. C., Luque R. (2014). Chem. Soc. Rev..

[cit29] Bueno-Alejo C. J., Hueso J. L., Mallada R., Julian I., Santamaria J. (2019). Chem. Eng. J..

[cit30] Bi W., Hu Y., Jiang N., Zhang L., Jiang H., Zhao X., Wang C., Li C. (2020). Appl. Catal., B.

[cit31] Deng Z., Ji J., Xing M., Zhang J. (2020). Nanoscale Adv..

[cit32] Xu J., Su X., Duan H., Hou B., Lin Q., Liu X., Pan X., Pei G., Geng H., Huang Y., Zhang T. (2016). J. Catal..

[cit33] Karelovic A., Ruiz P. (2013). J. Catal..

[cit34] Petala A., Panagiotopoulou P. (2018). Appl. Catal., B.

[cit35] Vogt C., Monai M., Kramer G. J., Weckhuysen B. M. (2019). Nat. Catal..

[cit36] Dalle K. E., Warnan J., Leung J. J., Reuillard B., Karmel I. S., Reisner E. (2019). Chem. Rev..

[cit37] Zhou R., Rui N., Fan Z., Liu C. J. (2016). Int. J. Hydrogen Energy.

[cit38] Kwak B. S., Vignesh K., Park N. K., Ryu H. J., Baek J. I., Kang M. (2015). Fuel.

[cit39] Meng A., Wu S., Cheng B., Yu J., Xu J. (2018). J. Mater. Chem. A.

[cit40] Billo T., Fu F.-Y., Raghunath P., Shown I., Chen W.-F., Lien H.-T., Shen T.-H., Lee J.-F., Chan T.-S., Huang K.-Y., Wu C.-I., Lin M. C., Hwang J.-S., Lee C.-H., Chen L.-C., Chen K.-H. (2018). Small.

[cit41] Chen W. T., Chan A., Sun-Waterhouse D., Moriga T., Idriss H., Waterhouse G. I. N. (2015). J. Catal..

[cit42] Chen W. T., Chan A., Sun-Waterhouse D., Llorca J., Idriss H., Waterhouse G. I. N. (2018). J. Catal..

[cit43] Lee Y., Kim E., Park Y., Kim J., Ryu W. H., Rho J., Kim K. (2018). J. Mater..

[cit44] Wenderich K., Mul G. (2016). Chem. Rev..

[cit45] Dadsetan S., Baghshahi S., Farshidfar F., Hadavi S. M. M. (2017). Ceram. Int..

[cit46] Rodríguez J. L., Valenzuela M. A., Pola F., Tiznado H., Poznyak T. (2012). J. Mol. Catal. A: Chem..

[cit47] Ahmed A. Y., Kandiel T. A., Ivanova I., Bahnemann D. (2014). Appl. Surf. Sci..

[cit48] Iliev V., Tomova D., Bilyarska L., Tyuliev G. (2007). J. Mol. Catal. A: Chem..

[cit49] Mills A., Valenzuela M. A. (2004). J. Photochem. Photobiol., A.

[cit50] Bueno-Alejo C. J., Arca-Ramos A., Hueso J. L., Santamaría J. (2020). Catal. Today.

[cit51] Yang H., Jin Z., Fan K., Liu D. D., Lu G. (2017). Superlattices Microstruct..

[cit52] Jiang X., Zhang Y., Jiang J., Rong Y., Wang Y., Wu Y., Pan C. (2012). J. Phys. Chem. C.

[cit53] Guo Y., Xiao L., Zhang M., Li Q., Yang J. (2018). Appl. Surf. Sci..

[cit54] Fujiwara K., Okuyama K., Pratsinis S. E. (2017). Environ. Sci.: Nano.

[cit55] Irie H., Miura S., Kamiya K., Hashimoto K. (2008). Chem. Phys. Lett..

[cit56] Wei S., Wu R., Xu X., Jian J., Wang H., Sun Y. (2016). Chem. Eng. J..

[cit57] Lin X., Lin L., Huang K., Chen X., Dai W., Fu X. (2015). Appl. Catal., B.

[cit58] Lin W., Cheng H., He L., Yu Y., Zhao F. (2013). J. Catal..

[cit59] Van Doorslaer S. and Murphy D. M., EPR Spectroscopy in Catalysis, in EPR Spectrosc. Appl. Chem. Biol., ed. M. Drescher and G. Jeschke, Springer, Berlin, Heidelberg, 2012, pp. 1–39 10.1007/128_2011_237 21928011

[cit60] Morra E., Giamello E., Chiesa M. (2017). J. Magn. Reson..

[cit61] Hashem M., Saion E., Al-Hada N. M., Kamari H. M., Shaari A. H., Talib Z. A., Paiman S. B., Kamarudeen M. A. (2016). Results Phys..

[cit62] Naldoni A., Altomare M., Zoppellaro G., Liu N., Kment Š., Zbořil R., Schmuki P. (2019). ACS Catal..

[cit63] Jia T., Zhang J., Wu J., Wang D., Liu Q., Qi Y., Hu B., He P., Pan W., Qi X. (2010). Mater. Lett..

[cit64] Hurum D. C., Agrios A. G., Gray K. A., Rajh T., Thurnauer M. C. (2003). J. Phys. Chem. B.

[cit65] Biesinger M. C., Payne B. P., Grosvenor A. P., Lau L. W. M., Gerson A. R., Smart R. S. C. (2011). Appl. Surf. Sci..

[cit66] Biju V., Abdul Khadar M. (2002). J. Nanopart. Res..

[cit67] McIntyre N. S., Johnston D. D., Coatsworth L. L., Davidson R. D., Brown J. R. (1990). Surf. Interface Anal..

[cit68] Oi L. E., Choo M. Y., Lee H. V., Ong H. C., Hamid S. B. A., Juan J. C. (2016). RSC Adv..

[cit69] Xu Z., Li Y., Zhang J., Chang L., Zhou R., Duan Z. (2001). Appl. Catal., A.

[cit70] Caballero A., Holgado J. P., Gonzalez-De la Cruz V. M., Habas S. E., Herranz T., Salmeron M. (2010). Chem. Commun..

[cit71] Gonzalez-De la Cruz V. M., Holgado J. P., Pereñíguez R., Caballero A. (2008). J. Catal..

[cit72] Wang R., Li Y., Shi R., Yang M. (2011). J. Mol. Catal. A: Chem..

[cit73] Zhao J., Liu B., Meng L., He S., Yuan R., Hou Y., Ding Z., Lin H., Zhang Z., Wang X., Long J. (2019). Appl. Catal., B.

[cit74] Hoch L. B., O'Brien P. G., Jelle A., Sandhel A., Perovic D. D., Mims C. A., Ozin G. A. (2016). ACS Nano.

[cit75] Stangeland K., Kalai D., Li H., Yu Z. (2017). Energy Procedia.

[cit76] Heine C., Lechner B. A. J., Bluhm H., Salmeron M. (2016). J. Am. Chem. Soc..

[cit77] Vogt C., Groeneveld E., Kamsma G., Nachtegaal M., Lu L., Kiely C. J., Berben P. H., Meirer F., Weckhuysen B. M. (2018). Nat. Catal..

[cit78] Abelló S., Berrueco C., Montané D. (2013). Fuel.

[cit79] Vogt C., Monai M., Sterk E. B., Palle J., Melcherts A. E. M., Zijlstra B., Groeneveld E., Berben P. H., Boereboom J. M., Hensen E. J. M., Meirer F., Filot I. A. W., Weckhuysen B. M. (2019). Nat. Commun..

[cit80] Lira E., Wendt S., Huo P., Hansen J. Ø., Streber R., Porsgaard S., Wei Y., Bechstein R., Lægsgaard E., Besenbacher F. (2011). J. Am. Chem. Soc..

[cit81] Ye J., He J., Wang S., Zhou X., Zhang Y., Liu G., Yang Y. (2019). Sep. Purif. Technol..

