# Supplementary material for: LED-driven controlled deposition of Ni onto TiO2 for visible-light expanded conversion of carbon dioxide into C1–C2 alkanes
Source: Nanoscale Adv. 2021 Apr 20;3(13):3788–98. doi: 10.1039/d1na00021g (PMC9417592; doi:10.1039/d1na00021g)
Supplement: NA-003-D1NA00021G-s001 [file NA-003-D1NA00021G-s001.pdf]

Electronic Supplementary Material (ESI) for Nanoscale Advances.
This journal is © The Royal Society of Chemistry 2021

Supplementary Information

LED-driven controlled deposition of Ni onto TiO_2 for the visible-light expanded conversion of carbon dioxide into C_1-C_2 alkanes.

A. Sanz-Marco,[1,2] J. L. Hueso,[1,2,3] V. Sebastian[1,2,3], D. Nielsen[4], S. Mossin[4], J.P. Holgado[5], C. J. Bueno-Alejo[1,2], F. Balas,[1,2,3*] and J. Santamaria[1,2,3*]

[1] *Department of Chemical and Environmental Engineering, C/Poeta Mariano Esquillor, s/n; Campus Rio Ebro, Edificio I+D, 50018, Zaragoza (Spain).*

[2] *Institute of Nanoscience of Aragon (INA)– University of Zaragoza, and Instituto de Ciencia de Materiales de Aragón (ICMA – CSIC); C/ Mariano Esquillor, s/n. 50018 Zaragoza (Spain).*

[3] *Networking Research Center in Biomaterials, Bioengineering and Nanomedicine (CIBER-BBN); C/ Monforte de Lemos, 3-5. 28029 Madrid (Spain).*

[4] *Centre for Catalysis and Sustainable Chemistry, Department of Chemistry, Technical University of Denmark, Kemitorvet 207, 2800 Kgs. Lyngby (Denmark)*

[5] *Instituto de Ciencia de Materiales de Sevilla (ICMS, CSIC-University of Seville), Avda. Americo Vespucio, s/n, 41092, Seville (Spain).*

EPR Operando protocol

The following protocol was performed on both P25 and Ni/TiO_2 to compare the response of the materials to UV light and to different gas mixtures.

The sample was first activated in 30% H_2 at 250°C. Then the influence of UV light and of the H_2/CO_2 gas (separately and together) was investigated at room temperature. Then the sample was reactivated and the procedure was repeated at 250 °C.

In general the spectra had lower resolution at 250 °C and changes in the spectra were more difficult to distinguish from the noise level. Overall the same evolution was observed, but less clear and only room temperature spectra are shown in the text. The spectrum assigned to Ni species (Type 1, see main text) changed with temperature.

The protocol performed on both P25 and on Ni/TiO_2 are given below. Small changes in time intervals have been ignored when making the table.

Table S1. EPR protocol for the *Operando* characterization of P25 and Ni/TiO_2 catalyst.

Event	Flow [ml/min]	T [°C]
30% H_2 on	15/35 H_2/He	Room temperature
Heat (15 min)	15/35 H_2/He	Ramp to 250°C by 15°/min
Activation in H_2(40 min)	15/35 H_2/He	250°C
Cool to RT(10 min)	15/35 H_2/He	Cool to RT
Light applied(100 s)	15/35 H_2/He	Room temperature
H_2+CO_2 on(10 min)	4/16/30 CO_2/H_2/He	Room temperature
Light applied(100 s)	4/16/30 CO_2/H_2/He	Room temperature
H_2+CO_2 off	4/16/30 CO_2/H_2/He	Room temperature
30% H_2 on	15/35 H_2/He	Room temperature
Heat (15 min)	15/35 H_2/He	Ramp to 250°C by 15°/min
Activation in H_2(5 min)	15/35 H_2/He	256°C
Light applied(100 s)	15/35 H_2/He	256°C
H_2+CO_2 on(10 min)	4/16/30 CO_2/H_2/He	256°C
Light applied(100s)	4/16/30 CO_2/H_2/He	256°C
H_2+CO_2 off, H_2 on	15/35 H_2/He	256°C
Cool to RT(10 min)	15/35 H_2/He	Cool to RT
H_2 off	50 He	Room temperature

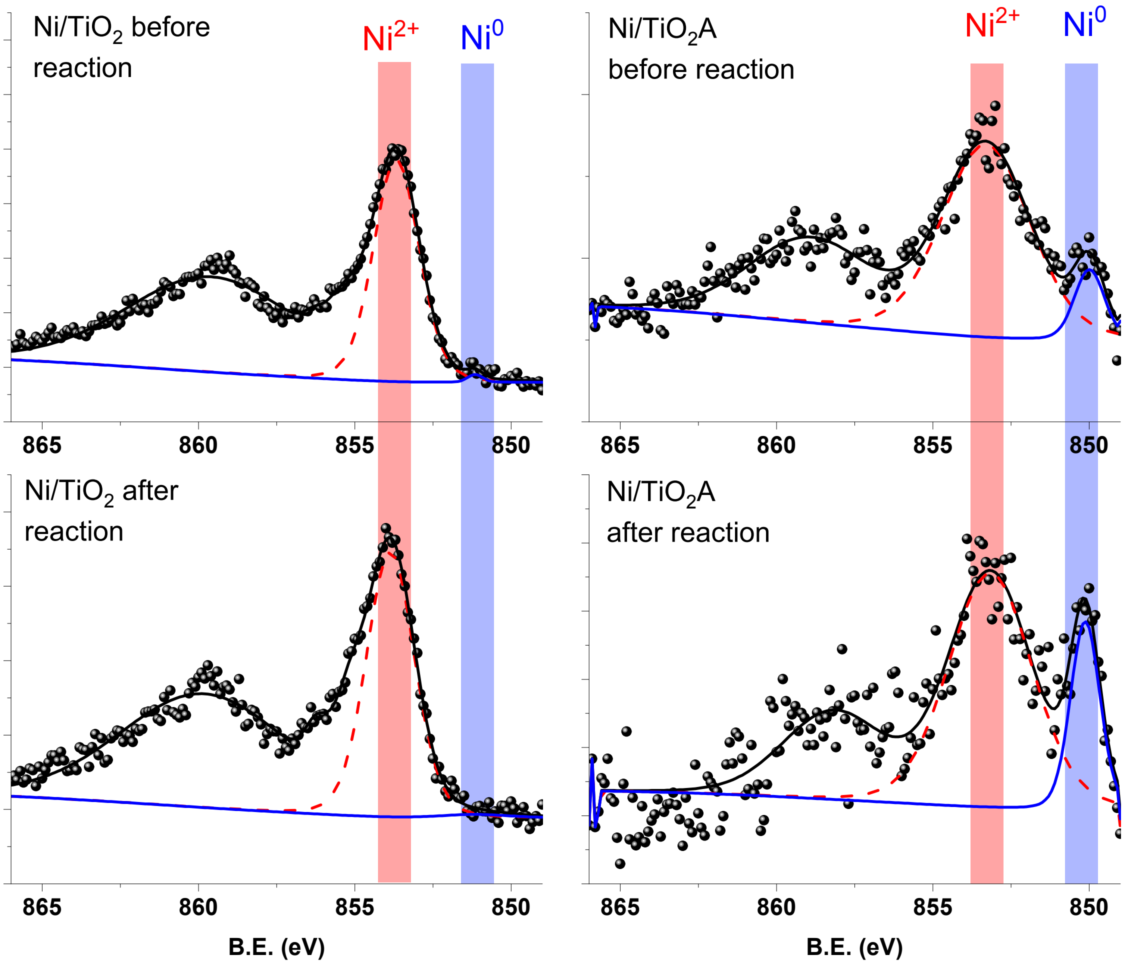

Figure S1: Detail of the XPS fitting parameters employed to calculate Ni(0)/Ni(II) ratios in the Ni2p3/2 regions registered in the in situ XPS measurements.

Table S2. Main parameters of Gaussian/Lorentzian peaks used for Ni2p fitting corresponding to Ni^0 and Ni^{2+} shapes.

Component for Ni2p Fittings		B. Energy / eV	FWMH	Areas / Ratio
Ni(II) component	Ni Oxidized-1	856.2	4.01	1.0
	Ni Oxidized-2	862.0	5.13	0.5
	Ni Oxidized-3	855.4	2.04	0.15
Ni^0 component	Ni Metallic-1	852.3	2.55	1.0
	Ni Metallic-2	856.9	3.31	0.18

Table S3. Percentages of Ni and Ti observed by XPS as determined by Ni2p/Ti2p corrected areas and ratio of Ni^0 and Ni^{2+} as determined by fitting using parameters of Table 1.

Spectrum	Ni2p %	Ti2p %	Ni^0 %	Ni^{2+} %
Original	7.1	92.9	3	97
H_2 400 ºC	1.6	98.4	57	43
CO_2+H_2 225 ºC	4.3	95.7	16	84
H_2 450 ºC	2.2	97.8	45	54
CO_2+H_2 225 ºC	2.4	97.6	49	51

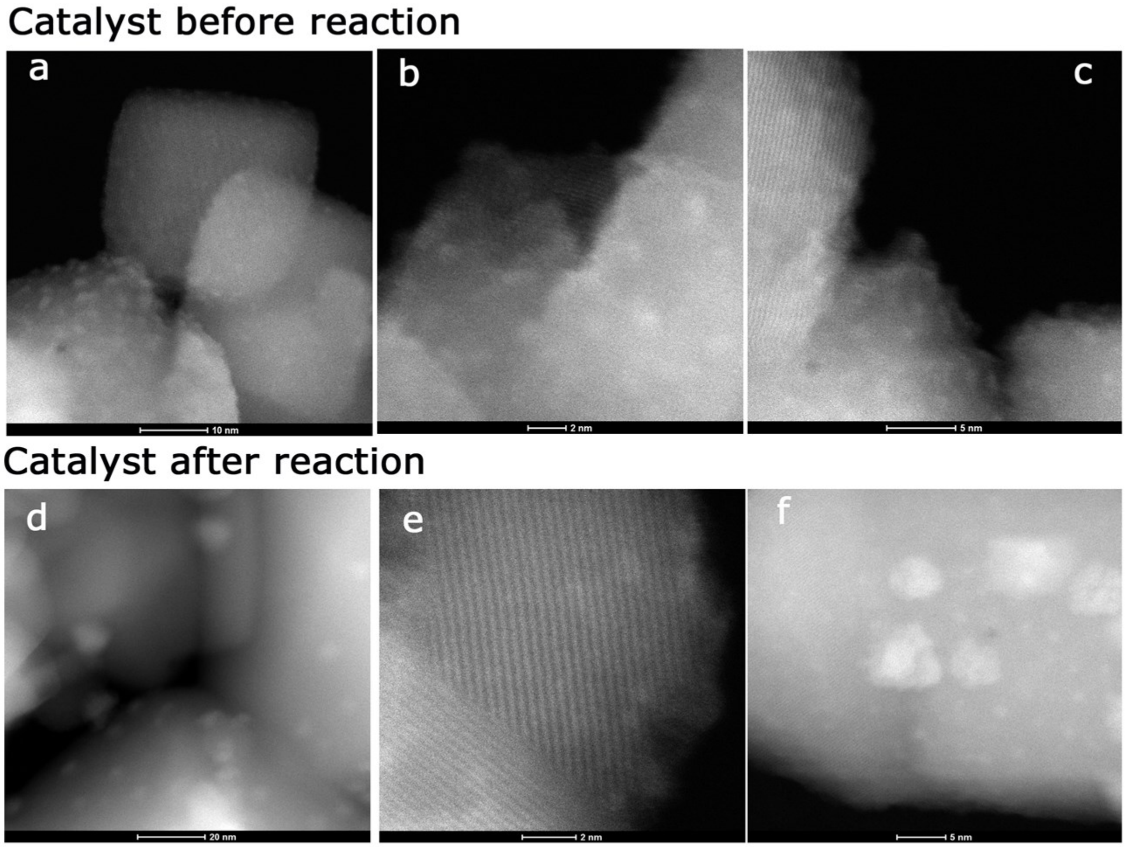

Figure S2. Additional HAADF-STEM representative images of the Ni/TiO_2: (a-c) before reaction; (d-f): after reaction.

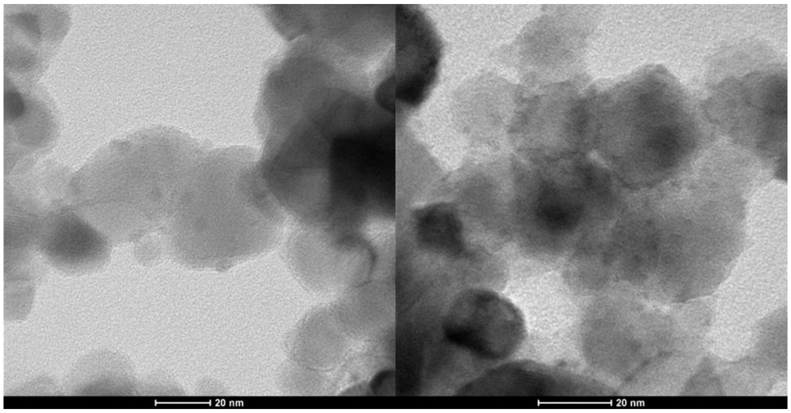

Figure S3. Additional TEM images of the Ni/TiO_2-A catalyst after photocatalytic hydrogenation under LED irradiation at 365 nm

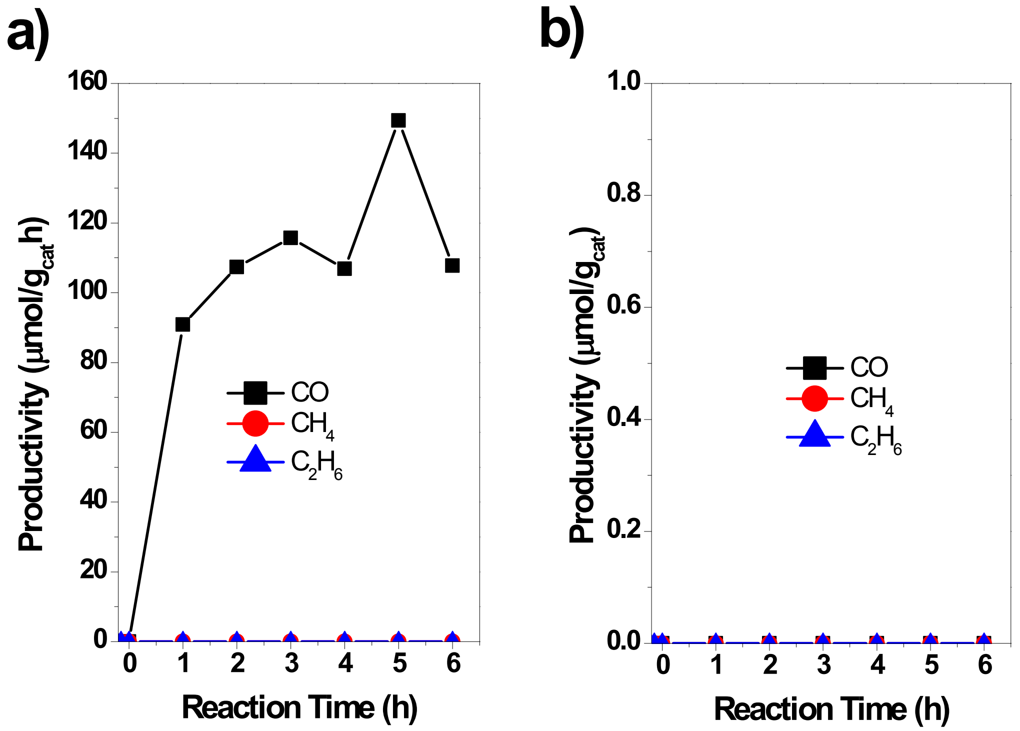

Figure S4. Productivity *vs.* time plots for P25 (a) under LED light irradiation at 365nm and for Ni/TiO_2 under LED light irradiation at 365nm and H_2:Ar (1:4) gas mixture.
